# Fluid Therapy: Double-Edged Sword during Critical Care?

**DOI:** 10.1155/2015/729075

**Published:** 2015-12-22

**Authors:** Jan Benes, Mikhail Kirov, Vsevolod Kuzkov, Mitja Lainscak, Zsolt Molnar, Gorazd Voga, Xavier Monnet

**Affiliations:** ^1^Department of Anesthesia and Intensive Care Medicine, University Teaching Hospital and Faculty of Medicine in Plzen, Charles University Prague, Alej Svobody 80, 32300 Plzen, Czech Republic; ^2^Department of Anesthesiology and Intensive Care Medicine, Northern State Medical University, Arkhangelsk 163000, Russia; ^3^Department of Cardiology and Department of Research and Education, General Hospital Celje, Oblakova 5, SI-3000 Celje, Slovenia; ^4^Faculty of Medicine, University of Ljubljana, Vrazov trg 2, SI-1000 Ljubljana, Slovenia; ^5^Department of Anaesthesiology and Intensive Therapy, Faculty of Medicine, University of Szeged, 6 Semmelweis Street, Szeged 6725, Hungary; ^6^Medical ICU, General Hospital Celje, Oblakova 5, SI-3000 Celje, Slovenia; ^7^Medical ICU, Paris-Sud University Hospitals, Inserm UMR-S 999, Paris-Sud University, 94 270 Le Kremlin-Bicêtre, France

## Abstract

Fluid therapy is still the mainstay of acute care in patients with shock or cardiovascular compromise. However, our understanding of the critically ill pathophysiology has evolved significantly in recent years. The revelation of the glycocalyx layer and subsequent research has redefined the basics of fluids behavior in the circulation. Using less invasive hemodynamic monitoring tools enables us to assess the cardiovascular function in a dynamic perspective. This allows pinpointing even distinct changes induced by treatment, by postural changes, or by interorgan interactions in real time and enables individualized patient management. Regarding fluids as drugs of any other kind led to the need for precise indication, way of administration, and also assessment of side effects. We possess now the evidence that patient centered outcomes may be altered when incorrect time, dose, or type of fluids are administered. In this review, three major features of fluid therapy are discussed: the prediction of fluid responsiveness, potential harms induced by overzealous fluid administration, and finally the problem of protocol-led treatments and their timing.

## 1. Introduction

In patients with acute circulatory failure, the primary goal of volume expansion is to increase cardiac output, hence oxygen delivery to the tissues. However, this effect is inconstant [1]: in many instances, fluid administration does not result in any hemodynamic benefits. In such cases, fluids may exert deleterious effects. In this regard, it is now well demonstrated that excessive fluid administration is associated with increased mortality, especially during acute respiratory distress syndrome (ARDS) [2] and in sepsis or septic shock [3, 4]. Whether this association between increased mortality and fluid accumulation is only an epiphenomenon based on illness severity or whether fluids exert harmful effect* per se* has to be further elucidated. However, in reality both features may contribute in part to what makes adequate fluid administration even more important. This is in contrast with the rather benevolent and uncoordinated use of fluids by clinicians worldwide as demonstrated in the recent FENICE trial [5]. In this review, we will focus on three major features of fluid therapy, which can be regarded as a double-edged sword: the prediction of fluid responsiveness, potential harms induced by overzealous fluid administration, and finally the problem of protocol-led treatments and their timing.

## 2. Predicting Fluid Responsiveness

The risks associated with improper fluid administration led to development of several strategies to assess “fluid responsiveness” before performing volume expansion. Although conventional parameters of preload have been used for decades for testing fluid responsiveness, their unreliability has been demonstrated by several studies [6]. Therefore, “dynamic” indices have been introduced in order to replace these unreliable “static” markers of preload. These dynamic indices are based on the changes in cardiac output or stroke volume resulting from changes in preload, induced by mechanical ventilation, by postural maneuvers, or by the infusion of small amounts of fluids [7]. In this chapter, we will describe the advantages and drawbacks of these “dynamic” indices of fluid responsiveness and the clinical setting where they may be applicable.

### 2.1. Static Indices of Cardiac Preload

**Figure 1:**
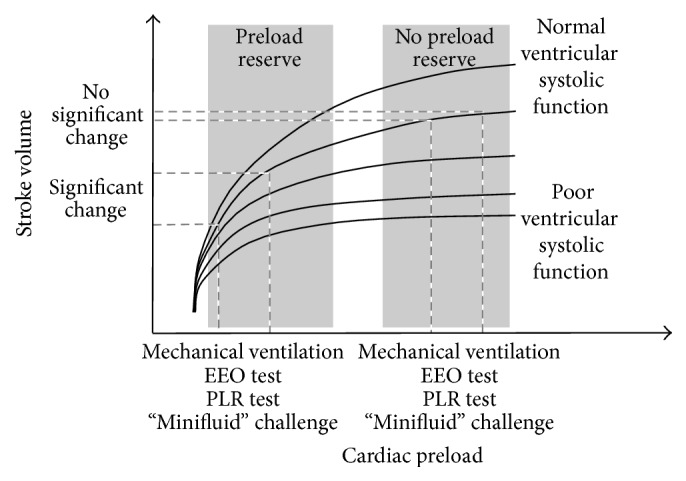
Cardiac function curve. There is a family of cardiac function curves depending on the ventricular contractility. If the ventricles are functioning on the steep part of cardiac function curve, changes in cardiac preload induced by mechanical ventilation, end-expiratory occlusion (EEO), passive leg raising (PLR), or “mini fluid challenge” result in significant changes in stroke volume. This is not the case if the ventricles are functioning on the steep part of cardiac function curve.

It is today clearly established that static markers of cardiac preload, such as the central venous pressure or pulmonary artery occlusion pressure, are unable to predict what effect will fluid administration have on cardiac output [6, 8]. The main explanation comes from basic physiology. Indeed, the slope of the cardiac function curve depends on the cardiac systolic function (Figure 1). Since this slope is unknown in a given patient, an absolute value of any “static” measure of preload could correspond to preload dependence and to preload independence. Another explanation for the unreliability of static markers of preload comes from the errors that can occur in their measurements. For instance, the measurement of central venous pressure requires a precise positioning of the pressure transducer with respect to the right atrium. It must also be carefully measured at end-expiration and should take into account the transmission of intrathoracic pressure to the right atrium. Similarly, the pulmonary artery occlusion pressure suffers from many possible errors in its measurement and interpretation [9]. To address shortcomings of these static indices, alternative methods have been developed to predict preload responsiveness. They fit in an overall concept of functional hemodynamic monitoring. They consist in observing some changes in cardiac preload, induced by mechanical ventilation, leg raising, or fluid challenges and in observing the resultant change of cardiac output or stroke volume [10].

### 2.2. Variations of Stroke Volume Induced by Mechanical Ventilation

#### 2.2.1. Physiological Background

During mechanical ventilation, insufflation increases the intrathoracic pressure, increases the right atrial pressure, and hence decreases the pressure gradient of venous return. If the right ventricle is preload-dependent, this will inevitably reduce the right ventricular outflow. Increase in right ventricular afterload induced by increased lung volume contributes to this reduction of right ventricular outflow during inspiration. As certain time is needed for the transit of blood through the pulmonary vasculature, this will reduce left ventricular preload. During conventional ventilation, this should occur at expiration. If the left ventricle is preload-dependent, the left ventricular stroke volume transiently decreases at expiration. Hence, a cyclic variation of stroke volume under mechanical ventilation indicates preload-dependence of both ventricles [11].

#### 2.2.2. How to Assess the Respiratory Variations of Stroke Volume?

Several surrogates or estimates of stroke volume have been used in order to quantify these respiratory variations. The first one was the systemic arterial pulse pressure [12], which is proportional to stroke volume. A number of studies have actually demonstrated that pulse pressure variation (PPV) is a reliable indicator of fluid responsiveness, provided that the conditions of its validity are fulfilled. The large number of these studies and a positive meta-analysis [13] contributed to establishment of a large and solid base of evidence for this indicator. Overall, the cut-off above which PPV is considered as significantly associated with fluid responsiveness is around 13%. Of course, as for many tests, this is not a strict cut-off. The farther from 13% the PPV value is, the higher is its diagnostic power.

Alongside PPV, other estimates of stroke volume have been used to predict preload responsiveness through their respiratory variations: stroke volume estimated by pulse contour analysis, blood flow of the left ventricular outflow tract measured by echocardiography, aortic blood flow assessed by esophageal Doppler, and the amplitude of the plethysmography signal recorded by pulse oximetry [1].

#### 2.2.3. Conditions of Validity, Advantages, and Limitations

The respiratory variation of stroke volume as a marker of preload responsiveness is not valid under some conditions. First, in case of spontaneous breathing activity, stroke volume variations relate more to the respiratory irregularity compared to preload dependence [14]. Second, in case of cardiac arrhythmias, the variation of stroke volume within the respiratory cycles is obviously more related to arrhythmia itself than to heart-lung interactions. The third important limitation refers to ARDS [15]. In such cases, low tidal volume and/or low lung compliance [16], which reduces the transmission of changes in alveolar pressure to the intrathoracic structures, both can diminish the amplitude of the ventilation-induced changes of intravascular pressure. This may result in false negative predictions of fluid responsiveness by PPV. Open chest surgery due to the low ratio of heart rate over respiratory rate [17] (corresponding in fact to respiratory rates at 40 breaths/minute or more) and intra-abdominal hypertension [18] are other circumstances in which PPV is unreliable to predict fluid responsiveness [10]. Overall, the limitations to the use of PPV are much more frequently encountered in the intensive care unit than in the operating theatre [19, 20].

#### 2.2.4. Respiratory Variation of Venae Cavae

Mechanical ventilation could induce some changes in the diameter of venae cavae. Due to their high compliance, the changes are more likely to be observed in case of hypovolemia than in case of normo- or hypervolemia. The respiratory variation of the diameter of the inferior vena cava at the point where it enters the thorax was demonstrated to reliably predict fluid responsiveness [21]. This was also the case for the collapsibility of the superior vena cava [22]. The most important limitation of these methods is that they have only limited predictive value in case of spontaneous breathing activity [23] mainly because of inhomogeneous respiratory efforts. As for PPV, low lung compliance and mechanical ventilation with a low tidal volume should theoretically minimize the effect of ventilation on the vena cava diameter and may thus invalidate the method. By contrast, these methods can be used in case of cardiac arrhythmias. The respiratory variation of the inferior vena cava is simple to measure by transthoracic echocardiography, which represents an important advantage. This could be particularly useful at the early phase of care, when arterial cannulation is yet to be done. The collapsibility of the superior vena cava is much more difficult to measure and requires transesophageal echocardiography. In a patient equipped with an arterial catheter, it is easier to use PPV than the superior vena cava collapsibility.

### 2.3. The End-Expiratory Occlusion (EEO) Test

#### 2.3.1. Hemodynamic Effects

As stated above, during the mechanical ventilatory cycle cardiac preload is reduced in inspiration. Stopping mechanical ventilation at end-expiration for a few seconds interrupts this cyclic decrease, meaning that end-expiratory occlusion (EEO) induces a transient increase in cardiac preload. This increase allows testing preload dependence. If the right ventricle is preload-dependent, the EEO will lead to an increased right ventricular output. If the duration of EEO is long enough for the transmission of this increased output toward the left cardiac cavities through the pulmonary circulation, left ventricular preload will increase. If the left ventricle is preload-dependent, EEO will eventually provoke an increase in cardiac output (Figure 1). Some studies consistently showed that if cardiac output increases by more than 5% during a 15-second EEO test, volume responsiveness could be predicted with a good reliability [16, 24].

#### 2.3.2. Advantages and Limitations

**Figure 2:**
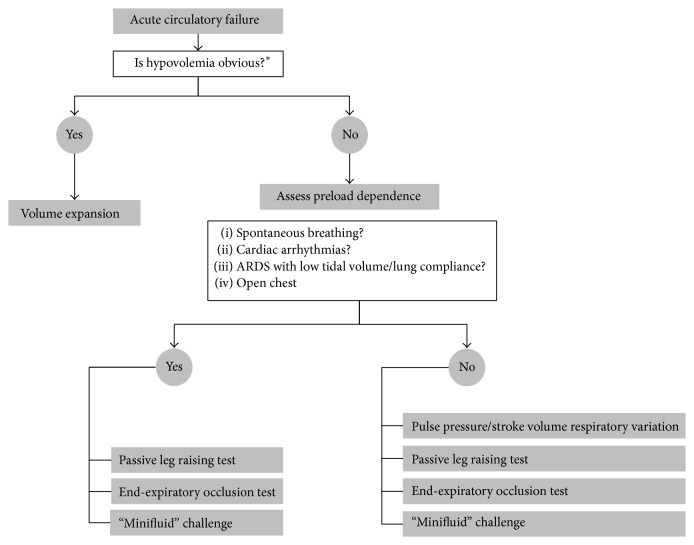
Decision-making algorithm of fluid administration. ^*∗*^Very initial phase of septic shock, when no fluid has been administered yet: in case of haemorrhagic shock or in case of hypovolemic shock due to diarrhoea, vomiting, or ketoacidosis, for instance.

Beyond its simplicity, an advantage of the EEO test is that it can be used in case of cardiac arrhythmias since it exerts its effects on a period of time (15 sec) that covers several cardiac cycles [24] (Figure 2). The EEO test can be used in patients who are not fully paralyzed and deeply sedated, unless a too marked triggering activity interrupts the 15-second EEO. Another limitation of the EEO test is that it is much easier to assess with a real-time measurement of cardiac output, such as pulse contour analysis [24]. Even if the increase in arterial pulse pressure during EEO is also indicative of fluid responsiveness [24], it requires either printing the arterial pressure curve or displaying the arterial pressure curve with a large scale, what is not allowed by many standard bedside monitors.

The EEO test remains valid whatever the level of positive end-expiratory pressure. One study reported that the prediction of fluid responsiveness by the EEO test was reliable and similar if the positive end-expiratory pressure was either 5 cmH_2_O or 13 cmH_2_O [25].

### 2.4. Fluid Challenge

The most intuitive way to test fluid responsiveness is to administer a small volume of fluid, observe its effects on cardiac output, and expect that a subsequent larger volume expansion will exert similar effects (Figure 1). The question is what should be considered as a “small” volume of fluid. The disadvantage of the “common” fluid challenge is that it consists of infusing 300–500 mL of fluid [26]. This volume is far to be negligible. Indeed, performing the fluid challenge several times a day, as it can be necessary at the early phase of shock, inevitably leads to a significant total volume of fluid that contributes to fluid overload.

A “mini fluid challenge” has been described as an alternative [27]. In an interesting study, the effects of only 100 mL of colloid on stroke volume predicted the response of cardiac output to a 500 mL volume expansion. These changes in stroke volume were estimated by echocardiography [27]. Nevertheless, small amounts of fluid can only induce small changes in stroke volume and cardiac output. Thus, this test requires a very precise technique for measuring cardiac output. Whether echocardiography is precise enough in nonexperts' hands is far to be certain. The interest of more precise measurements of cardiac output will likely be investigated.

### 2.5. The Passive Leg Raising (PLR) Test

#### 2.5.1. Hemodynamic Effects

In a patient lying in the semirecumbent position, elevating the inferior limbs at 45° and lowering the trunk induces a transfer of venous blood from the lower part of the body toward the cardiac cavities. PLR increases right and left cardiac preload and acts like a transient and reversible “self-volume challenge” [28] (Figure 1). The reliability of PLR as a test of preload responsiveness has been demonstrated by several studies. An increase of cardiac output above approximately 10% predicts the response to a subsequent volume expansion with good sensitivity and specificity [29]. A recent meta-analysis of all studies performed with the PLR test confirms its strong reliability [30].

#### 2.5.2. Advantages, Limitations, and Technical Considerations

Since its hemodynamic effects occur over a long period of time, PLR remains valid in case of cardiac arrhythmias and spontaneous breathing activity. The hemodynamic effects of PLR are independent from mechanical ventilation, which explains that the PLR test can be used in some conditions in which PPV is not valid, such as low tidal volume, low lung compliance, and very high respiratory rate (Figure 2). The postural change used for PLR is important to consider. Firstly, the test should start from the semirecumbent and not from the horizontal supine position [33]. Indeed, if it starts from the semirecumbent position, PLR includes the lowering of the trunk, which is associated with a transfer of venous blood from the large splanchnic compartment to the cardiac chambers. It was actually demonstrated that this technique exerts larger hemodynamic effects compared to a horizontal starting position [34]. Secondly, it is important to move the bed of the patient and not the patient itself, because sympathetic stimulations induced by passive hip movement and pain may invalidate the measurement [33]. In this regard, the absence of an increase in heart rate during the PLR test should be checked as it shows that the changes in cardiac output are not related to sympathetic stimulation.

Another important point regards the method that must be used for assessing the PLR-induced hemodynamic changes [33]. Firstly, these effects cannot be assessed by observing the simple arterial pressure. Indeed, it has been demonstrated that the PLR-induced changes in arterial pulse pressure unreliably predict preload responsiveness, with a significant number of false negatives [29]. This is most likely due to the fact that PLR modifies the physiological properties of the arterial tree, thus changing the relationship between arterial pulse pressure and stroke volume. Thus, a technique that directly measures cardiac output is mandatory [33]. Secondly, the effects of PLR must be assessed with a technique providing a real-time measurement of cardiac output. The maximal effect on cardiac output usually occurs within one minute [29]. In some patients with a strong vasodilatation and capillary leak, the effects of PLR progressively vanish over a few minutes. This explains why pulmonary or transpulmonary thermodilution techniques, which take tens of seconds to repeat cold fluid boluses, are not suitable. The PLR method has been tested by esophageal Doppler and the changes in aortic blood flow, with pulse-contour analysis and the changes in cardiac output, cardiac output measured by bioreactance and endotracheal bioimpedance cardiography, subaortic blood velocity measured by echocardiography and ascending aortic velocity measured by suprasternal Doppler [33]. Interestingly, in patients on mechanical ventilation with perfectly regular ventilation, the PLR-induced changes in cardiac output could be simply and noninvasively be estimated by the changes in end-tidal carbon dioxide [35, 36]. This should allow using the PLR test in the absence of any cardiac output monitoring device.

Intra-abdominal hypertension could reduce the validity of the PLR test. It has been suggested that intra-abdominal hypertension could create an obstacle to the transfer of blood from the lower limbs toward the cardiac chambers through the inferior vena cava [37]. One study suggested that the PLR test was not reliable anymore if the intra-abdominal pressure was higher than 16 mmHg [38]. Nevertheless, this study did not measure the intra-abdominal pressure during PLR. Thus, this possible limitation of the PLR test needs further confirmation.

### 2.6. Using Predictors of Fluid Responsiveness in Practice

The prediction of fluid responsiveness should be considered differently upon the clinical setting. First, one must remember that preload dependence is a physiological condition. Thus, positive predictors of fluid responsiveness should lead to volume expansion only in case of circulatory failure. Second, in case of an obvious hypovolemia, detecting preload dependence is useless since fluid responsiveness is constant. This is the case at the very initial phase of septic shock, when no fluid has been already administered, in case of hemorrhagic shock or in case of hypovolemic shock due to diarrhea, vomiting, or ketoacidosis, for instance.

The operating theatre might be particularly adapted for the respiratory variation of stroke volume or surrogates in anesthetized patients, except if low tidal volumes are used for mechanical ventilation. In addition, such indices can be assessed by means of a simple arterial catheter or by noninvasive hemodynamic monitoring devices, which are suitable for this setting. The EEO test might also be useful, provided that the ventilator allows interrupting ventilation at end-expiration for 15 sec.

In critically ill patients, the frequent presence of cardiac arrhythmias, the low lung compliance and ventilation with low tidal volumes associated with ARDS, and the presence of some spontaneous breathing and of low lung compliance often prevent use of PPV and the related indices. The respiratory variation of vena cava can be used as an alternative in case of cardiac arrhythmias. The EEO and PLR tests are often suitable provided that their conditions of application are fulfilled.

The utility of predictors of fluid responsiveness may also depend upon the context where they are used. In the perioperative setting, prediction of fluid responsiveness might be part of the preemptive, individualized hemodynamic treatment that has been shown to reduce the rate of postoperative complications and the hospital length-of-stay in different categories of surgical patients [39–41]. In the context of intensive care, indicators of preload dependence may be particularly useful to differentiate between fluid responder and nonresponder patients, hence avoiding “underresuscitation” and/or “overresuscitation,” both of which are associated with poor prognosis in case of septic shock and ARDS [42].

## 3. The Risks of Fluid Therapy

**Figure 3:**
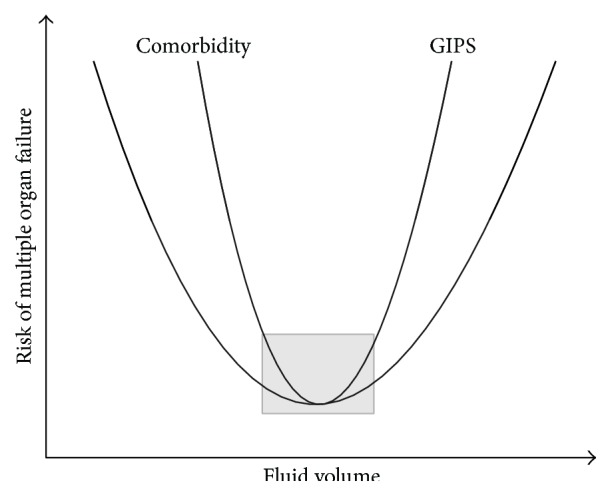
The risks of insufficient and excessive fluid resuscitation. GIPS—global increased permeability syndrome.

Today, we possess clear evidence of how detrimental unjustified and unbalanced fluid administration might be. Similar to several therapeutic interventions, fluid administration is obviously a life-saving intervention in severe hypovolemia and dehydration; however, it can also exert a number of adverse and potentially life-threatening effects (Figure 3). The effects of infusion therapy are determined by discovery of the novel mechanisms in fluid exchange. Thus, a recognition of two new important players, glycocalyx barrier and active water transporters (aquaporins), led to the critique of the Starling concept [43, 44]. The pathophysiological findings of the shift from intravascular to subglycocalyx oncotic pressure resulted in substantial change of our knowledge of the process of vascular fluid transport [45, 46]. Currently, we must realize that fluid distribution within the body of critically ill patients has become as unpredictable as ever. However, understanding these mechanisms during fluid therapy can be helpful to prevent its potential risks and follow the* primum nil nocere* principle.

### 3.1. “Third Hit” of Shock

A number of guidelines recommend an aggressive and early “rescue” fluid resuscitation, particularly in severe sepsis, hemorrhagic shock, and necrotizing pancreatitis [47–49]. The body of evidence has shown that fast repletion of fluid deficit in shock using crystalloid and/or colloid solutions within a period of first 3–24 hours after admission prevents the critical decrease of oxygen delivery, attenuates the severity of multiple organ dysfunctions, and reduces the incidence of adverse effects. However, everything has its price. The most common type of dysoxia in ICU, distributive shock, can be associated with delayed “flow” phase global increased permeability syndrome (GIPS) [50]. Under conditions of increased vascular permeability, mainly due to glycocalyx injury and disturbances of lymphatic flow, fluids are leaving vascular bed and expand the interstitium. This scenario results in total weight gain and edema formation, influencing the volumes and interstitial pressure in lungs, splanchnic viscera, and peripheral tissues. An increase of body weight by more than 10% compared with baseline during ICU stay confirms the hyperhydration and may result in secondary and, hence, delayed organ dysfunction—which may be referred to as “third hit” of shock [51, 52].

The primary aim of the fluid load is to increase cardiac output and oxygen delivery in patients with compromised oxygen transport. Thus, the dynamic parameters and functional hemodynamic tests can be of great value in determining the clinically effective volume of fluid load. However, in distributive shock associated with severe GIPS targeting “normal” preload and cardiac index can result in life-threatening complications. In severe ARDS, burns, or shock associated with necrotizing pancreatitis even restrictive fluid load can be accompanied by intense fluid accumulation in the tissues, particularly in the lungs, leading to increase in extravascular lung water (EVLW), hypoxemia, and pulmonary edema. This dilemma exerts a “therapeutic conflict” that forces us to modify the goal-directed intervention and consider “permissive hypovolemia” [53]. Without any doubts, this approach can be especially useful in the settings of advanced volumetric and metabolic monitoring.

### 3.2. Fluid Resuscitation or Accumulation

In the literature, we can find wide range of terms describing fluid therapy in both ICU and perioperative settings including “liberal,” “conservative,” and “restrictive.” In the review of Bundgaard-Nielsen et al. [54] merging results of seven major randomized studies, the volume of intraoperative “liberal” approach ranged from 2750 to 5388 mL, while “restrictive” approach was limited to 998–2740 mL. Therefore, the exact borders of these strategies are rather blurred, requiring individualized goal-directed titration of the fluid in most cases.

In the ICU, an excessive fluid load in the settings of GIPS, systemic inflammatory response syndrome, and multiple organ dysfunction, particularly, in ARDS and acute kidney injury (AKI), can be devastating (Table 1). The RENAL study has shown that negative net fluid balance is an independent predictor of reduced 90-day mortality and increased post-ICU lifespan [55]. In this study, the handling of negative fluid balance significantly decreased the duration of renal replacement therapy and length of ICU stay. Therefore, the goal of “negative fluid balance” should be widely adopted as important part of the delayed goal-directed therapy of critically ill patients. The spontaneous (Deescalation) or triggered (“Evacuation”) removal of excessive fluid becomes the link of the modern “chain of ICU survival”—“Rescue-Optimisation-Stabilisation-Deescalation/Evacuation” (ROSD/E, see Section 4.2) [51, 52]. One from the examples for such an approach represents goal-directed ultrafiltration resulting in attenuation of intraabdominal hypertension (guided by intra-abdominal pressure (IAP)), volume overload (guided by global end-diastolic index), and pulmonary edema (guided by EVLW) [56].

During the perioperative period, the liberal fluid therapy is commonly justified by presumption of perioperative dehydration and losses into hypothetical “third space.” Current evidence shows that in many patients these factors can hardly be assumed as an important reason for perioperative hemodynamic distress and should not be considered as a prerequisite for the aggressive intraoperative or preoperative fluid load [57]. Thus, in neuraxial anesthesia, vasodilatation caused by arterial hypotension is often treated with extensive fluid load; however, under these conditions the increase in preload does not counteract vasodilation and rarely increases systemic arterial pressure [58, 59]. Furthermore, several studies demonstrated that postoperative weight gain is associated with the risk of severe complications [60]. Along with other perioperative factors (ischemia-reperfusion, cytokine release, hyperoxia, etc.) excessive fluid load may be accompanied by release of atrial natriuretic peptide and the injury of the glycocalyx layer [61]. Moreover, in major abdominal surgery, liberal fluid therapy can pose the risk of increased IAP, respiratory complications, and delayed anastomosis leakage [62].

Therefore, the risks of fluid resuscitation can mainly be related to the following factors:

1. volume and rate of fluid administration (deliberate or excessive fluid load),
2. reperfusion phenomenon and microcirculatory recruitment,
3. fluid-specific complications (AKI for hydroxyethyl starches (HES) or dilutional acidosis for unbalanced crystalloids).

### 3.3. Choice of Fluid

The choice of type of fluid does probably not primarily affect the clinically important outcomes related to the adverse effects of hyperhydration. However, at later stages the effects of type of fluid become unequivocal. According to the range of the current guidelines the use of albumin and semisynthetic colloids does not carry any obvious benefits over crystalloids and can be harmful in particular subgroups of patients like traumatic brain injury [63]. The risks of acute kidney injury and coagulation disorders related to HES-administration are well recognized in numerous studies [64]. On the other side, some guidelines, for example, European Society of Anesthesiology, comment that, compared with crystalloids, hemodynamic stabilization using isooncotic colloids (albumin, HES) may decrease tissue edema (the quality of evidence, “C”) [48]. Indeed, if colloids are leaking from the vascular bed under settings of GIPS and glycocalyx flaking, they can hold the fluid within the interstitium at similar extent like within the intact vasculature.

Initial fluid resuscitation should routinely be started with crystalloid solutions. However, according to Marik [60], hyperhydration with unbalanced crystalloids can result in “Iatrogenic salt water drowning.” Beyond the particular problem of hyperhydration, high dose of crystalloids, particularly 0,9% NaCl, increases the net chloride load, resulting in hyperchloremia and hyperchloremic (dilutional) metabolic acidosis. These disturbances were claimed to increase the risk of AKI and mortality [65, 66]. Thus, implementing the protocols of early goal-directed therapy, the use of high doses of unbalanced crystalloids should be avoided. The rational approach might be based on the administration of balanced, “chloride-restricted” crystalloids or, probably, in some situations (like refractory shock and ARDS in the absence of AKI and coagulopathy), on the combination of crystalloids with limited (up to 15 mL/kg) volumes of colloids.

### 3.4. Chapter Underline

It is important to note that the goal-directed fluid therapy aiming to central venous pressure, as still required by Surviving Sepsis Campaign [47], in many situations can be dangerous due to number of reasons and should be avoided [31, 32, 60]. The detrimental effects of forced CVP increase can result in numerous complications presented in Table 2. Both insufficient and excessive fluid resuscitation can be detrimental for the organ function and result in deterioration of clinical outcome both in ICU patients and in major surgery. This important clinical dilemma can be resolved using up-to-date advanced methods of hemodynamic and metabolic monitoring and “phasic” approach to fluid management of critically ill patients.

## 4. Timing and Use of Protocols for Fluid Therapy

### 4.1. Protocols for Fluid Management

In previous chapters, we have demonstrated several measures to differentiate between patients who would or would not benefit from fluid administration or in fact require fluid removal. Consequences of inadequate or overzealous fluid administration were also discussed. From this point of view, it sounds rational to use strategies enabling individual titration of fluid balance which should be associated with better outcomes in critically ill patients. However, it seems that our praxis is very divergent [5, 67–69]. This may be due to controversies existing in the evidence supporting currently available fluid and/or hemodynamic management protocols. The two recent large multicenter trials ARISE [70] and PROCESS [71] in septic patients showed that protocol-led care (namely, early goal-directed therapy according to Rivers et al. [72]) was comparable to standard “do-what-you-want” treatment in severe sepsis and/or septic shock. The outcomes observed in patients managed using advanced hemodynamic monitoring (with no protocol) were comparable to those without also in other studies [73, 74]. In a recent Chinese study treatment according to transpulmonary thermodilution derived volumetric variables based protocol also showed no measurable benefit [75] although this study received serious criticisms as well [76, 77].

Even in the perioperative setting where numbers of single and multicenter trials have proved significant benefit of advanced hemodynamic monitoring guided management on morbidity, contradictory results have also been published recently. The largest study so far on this topic, the OPTIMISE trial [78], failed to prove its primary outcome of the composite 30-day moderate to major morbidity and mortality. In this last part of this paper we would try to shed some light on this disproportion and offer the reader a rational approach to the use of individualized protocols and hemodynamic monitoring tools.

### 4.2. Timing of Fluid Interventions (the ROSD/E Concept)

**Figure 4:**
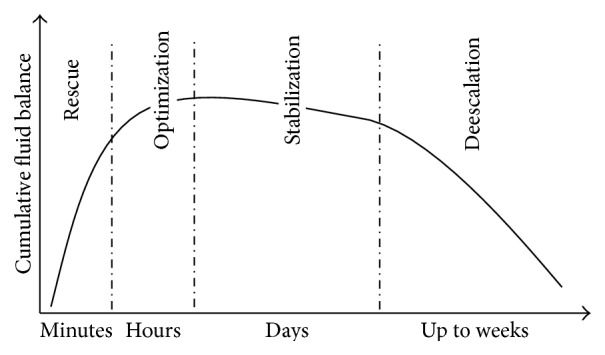
Four phases of hemodynamic treatment in relation to cumulative fluid balance.

It has been established in various scenarios that timing is crucial: immediate commencement of resuscitation and of the time within the target end-points reached is of utmost importance in critical care medicine. As recently pointed out by some most renowned authors [79, 80] four phases are distinguishable in the time course of critical illness: Rescue, Optimization, Stabilization, and Deescalation/Evacuation. In each of these four stages' treatment modalities, goals and monitoring tools will substantially differ (Figure 4, Table 3).

#### 4.2.1. Rescue

The Rescue phase encompasses the very first minutes to hours aiming at salvage of the patient. Naturally, only those readily available tools and minimalistic targets may be considered. In some cases, overtreatment may even be associated with harm as, for instance, overresuscitation to higher perfusion pressure with fluids in multiple trauma victims [81]. Evidence is lacking to support the use of protocols in this period. The EGDT/SSC protocol [47], namely, its first part (20 mL/kg bolus and further fluid and vasopressor administration to reach the MAP of 65 mmHg), may be considered as an example of resuscitation for patients with septic shock. However, there are some controversies regarding the predefined amount of fluid bolus, which varies from 20 mL/kg [71] to 40 mL/kg [82], but a single bolus of 1000 mLs has also been used [70]. One can also argue that considering the patients' individual premorbid conditions, higher or lower values of MAP may be more beneficial then what is recommended as a universal target in the guidelines. Reaching the lower autoregulatory threshold of the most vulnerable organs (heart and brain) seems to be the cornerstone. This is reflected in the usually proposed pressure targets: SAP of 80 mmHg (MAP 55 mmHg) in overall young and healthy population of trauma victims and MAP of 65 mmHg in septic patients mostly older with comorbidities. Besides standard pressure measurements, ultrasonography and echocardiography may offer valuable advanced information on heart function, including preload, contractility, and ventricular performance.

#### 4.2.2. Optimization

In the Optimization phase, the therapeutic goal should be to reach the optimal perfusion of peripheral tissue and, according to some authors, to repay the oxygen debt incurred through the previous course of acute illness [83]. It is necessary to emphasize that this phase seems to be time limited. Kern and Shoemaker [84] in their meta-analysis indicated that optimization of tissue perfusion should be done in the time-window of 24 hours after the insult, although physiological rationale suggests limiting oxygen debt for the shortest time possible. Studies trying to reach predefined oxygen delivery targets later failed to improve outcome [85, 86]. This time limit may prompt the use of protocols to assure appropriate care. Fluids, vasopressor, inotropes, and vasodilators are the most commonly used measures to reach optimization goals. Fluids are the mainstay, but in the case of decreased vascular tone vasopressors should be used as early as possible in order to minimize the pathological pooling of blood and help mobilizing the unstressed volume [87]. Providing inotropic support should be reserved for those patients who after optimizing both pre- and afterload still show unsatisfactory heart performance coupled with signs of organ hypoperfusion.

**Figure 5:**
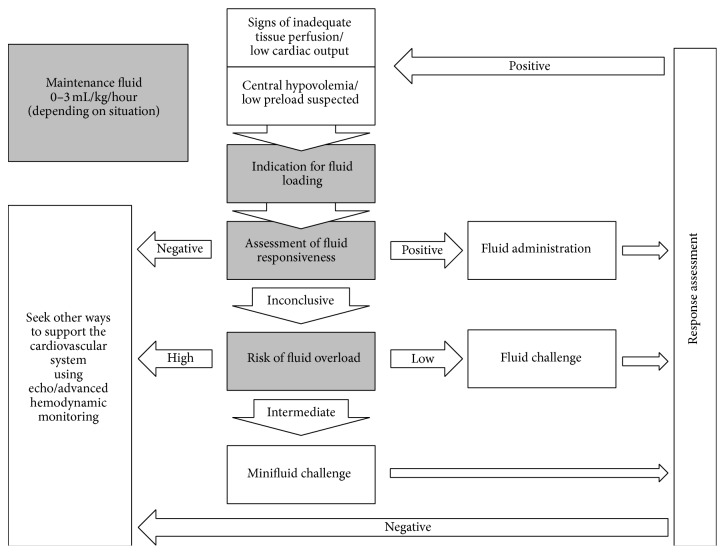
Decision algorithm for fluid loading in optimization phase.

Nevertheless, this pathophysiological rationale based approach is not supported by robust clinical data among critically ill patients, but several studies demonstrated benefit (length of stay and morbidity) in trauma victims [88, 89]. In severe sepsis, recent multicenter trials [70, 71, 75] proved that protocols based on targets with low predictive value for fluid responsiveness and local tissue perfusion (i.e., static parameters, CVP or ITBV, and continuous ScvO_2_) are comparable to consultant-led treatment alone. Similarly, there is no evidence supporting the early use of advanced hemodynamic monitoring. With widespread use of critical care echocardiography many information on the heart performance may be gathered with the use of a transthoracic probe and with standard monitoring (i.e., arterial pressure curve). More invasive tools of adequate reliability may help to manage difficult patients and also help less experienced physicians to understand the underlying physiology and follow treatment goals. In this view of low evidence, only general recommendation may be derived for the everyday care (Figure 5).

#### 4.2.3. Optimization in the Perioperative Setting

Perioperative goal-directed therapy (pGDT) has a special place in this topic of the protocol-guided therapy in the Optimization phase. In contrast to other critical care scenarios, both time and severity of the insult are well defined. Furthermore, the population of patients undergoing surgery is usually more homogenous from many aspects. Numerous studies and meta-analyses suggested that pGDT reduces the risk of postoperative complications [41, 90]. Still some controversy exists regarding the use of perioperative hemodynamic optimization. First, the rising use of less invasive hemodynamic monitoring devices led to widening of the indication. Nowadays less severe patients and procedures are considered suitable for pGDT as compared to previous years [40]. However, it follows simple logic that in these patients with reduced risks for perioperative complications only limited benefit may be expected. In fact the opposite may be true, as in a fit patient with better cardiopulmonary reserves; optimizing circulating volume to maximize the stroke volume may lead to unnecessary and potentially harmful positive fluid balance and accumulation [91]. Also, targeting the perioperative care to reach global preset values of oxygen delivery lacks advantage of individually tailored care and may be detrimental in some patients [92].

Nevertheless, surgical patients undergoing general anesthesia with controlled mechanical ventilation are ideal candidates for using dynamic predictors of fluid responsiveness (mostly based on heart-lung interactions) to rationalize intraoperative fluid therapy. Using stroke/pulse pressure volume variation or its surrogates was shown to be effective in reaching better outcomes and reducing postoperative complications in a recently published multicenter trial [93] as well as in a large meta-analysis [39]. Despite these promising results, dynamic variables have certain limitations. In a recent study by Cannesson et al., it was found that fluid responsiveness could not be predicted reliably and pressure variation was in the range of 9–13%, also called the “grey zone,” which is found in 24% of the cases undergoing surgery [94]. Regarding inotropes, until proved otherwise the use of inotropes should be limited for patients not achieving adequate stroke volume despite satisfactory preload and there are signs of suboptimal global tissue perfusion such as abnormal ScvO_2_, high lactate, and increased central venous-arterial CO_2_-gap. Adequate bedside tools and targets of regional tissue perfusion capable of monitoring microcirculation are, however, still undetermined.

#### 4.2.4. Stabilization and Deescalation/Evacuation

There is limited data regarding protocolized care in Stabilization and Deescalation. Aggressive initial treatment followed by a restrictive approach was demonstrated to be beneficial by Murphy et al. [95]. In ARDS patients FACTT [96] and recent FACTT lite trials [97] demonstrated that restrictive maintenance in stabilization phase was associated with improvement in outcome. It is of vital importance to recognize the right moment to stop the optimization, but unfortunately we possess no evidence based data to elucidate this critical moment. However, conventional indicators, such as the resolution of oliguria, a decrease in lactate levels, and improving ScvO_2_, can be helpful but may not occur in every patient. However, it is important to acknowledge that none of these indices have a high enough sensitivity on their own to be used as a target in every patient; therefore, the so-called “multimodal” approach, meaning to take all that into account, may be necessary. At any case, if the optimization phase lasts longer than 24 hours after the initial insult, it is not associated with improved outcomes.

Similar to the rather blurred borderline between Optimization and Stabilization phases the optimal moment for the initiation of Deescalation is also unresolved. In many patients the fluid “Deescalation” is a naturally occurring process as a result of the spontaneous healing, during which forcing fluid removal is unnecessary. However, in some cases either the positive fluid balance is too large or the ability of the patient to mobilize the edema is diminished by the disease and active intervention is necessary. For this reason, a “diuretic responsiveness test” was recently introduced [98], which may help to identify those patients being able to reach negative fluid balance by receiving diuretics. Achieving negative fluid balance by the combined administration of albumin and furosemide was shown to improve outcomes in a small trial [99] and in a retrospective study [100]. However, in some patients the response for diuretics remains poor. In these cases an active Deescalation by extracorporeal means, also termed “Evacuation,” may be considered. Nevertheless, the evidence is too weak to enable us to develop universally applicable protocolized treatment in these patients [101].

### 4.3. Chapter Underline

Cardiovascular insufficiency due to critical illness is a very heterogeneous entity. As pointed by Vincent et al. [102], patient populations are often defined as syndromes based on gross phenotypic variables (fever, tachycardia) rather than on distinct features of disease origin or chronic conditions. Mixing population of ARDS patients with septic ones may have altered the results of Zhang et al. study [75]. Severe sepsis due to pneumonia needs totally different approach to septic shock of intraabdominal origin in regard of fluid and hemodynamic treatment. These circumstances were probably recognized by an experienced clinician in the control arm of ARISE [70] or PROCESS [71] trials but may be neglected by protocol treatment.

## 5. Conclusions

Several tools are available today to assess volume responsiveness using dynamic procedures. These tools enable us to administer fluid with the assurance that it will lead to the expected increase in cardiac output. In particular, this should be included into the protocols guiding the hemodynamic treatment in the operating room setting. In the intensive care unit, these tools may be particularly useful in order to refrain from volume expansion that should reduce the risk of overzealous fluid administration. Further studies elucidating the kinetics of glycocalyx injury/regeneration and the role of vascular permeability during the goal-directed, individualized approach to the fluid resuscitation are warranted. The type and volume of the fluid should be thoroughly selected considering the phase of shock, risk of impending organ dysfunction, and individual comorbidity.

Failure to use the time-patient-tool-protocol adequate approach may lead to worse outcomes and false conclusions. Use of protocols without proper individualization will always offer simplistic solution and can never lead to improvement of care in all patients coming from some global population of different disease states and severity. In other words, protocols of care can never replace the well-educated and critically thinking physician, who is able choose the appropriate diagnostic tools, put all relevant data into context, and tailor treatment to the patients' individualized needs.

## Figures and Tables

**Table 1:** The risks of excessive fluid load.

Settings	Adverse effect	Comment
Perioperative	Hyperchloremia and dilutional acidosis	Can be reduced using anion-balanced crystalloid solutions
	Reduced rate of wound healing	Can be related to the peripheral tissue edema
	Increased risk of anastomosis leakage	Intestinal edema and decreased splanchnic perfusion
	Increased IAP	Intestinal and abdominal wall edema
	Increased risk of respiratory complications	Pulmonary and chest wall edema. Stressfully increased work of breathing

ICU	GIPS and glycocalyx injury	The decrease of subglycocalyx oncotic pressure facilitates the capillary leakage
	Increased IAP/ACS and polycompartment syndrome	Can be associated with polycompartment syndrome resulting in AKI, liver dysfunction, FRC reduction, and ileus
	Deranged oxygenation, pulmonary and chest wall edema, incidence, or increased ARDS severity	EVLWI increase. The fluid load is an independent risk factor of ARDS
	Enteropathy	Gut edema, bacterial translocation, malabsorption, and liver congestion
	Brain edema and increased ICP	Albumin is risky
	Kidney injury	Edema of kidney parenchyma with increase of *P*_INT_ and decreased GFR
	Myocardial injury	Dilatation, ANP release, and myocardium edema associated with diastolic dysfunction (relaxation) and blockade
	Increased mortality	

IAP: intraabdominal pressure, ICP: intracranial pressure, ACS: abdominal compartment syndrome, GIPS: global increased permeability syndrome, ANP: atrial natriuretic peptide, ARDS: acute respiratory distress syndrome, EVLWI: extravascular lung water index, and GFR: glomerular filtration rate.

**Table 2:** The risks of increased central venous pressure.

Consequence	Comment
Decreased venous return and cardiac index	CVP is not a reliable characteristic of preload and, when exceeding 8 mmHg, can be an independent predictor of the mortality [31]. The normal CVP value is close to 0. According to Guyton model, both venous return and cardiac output are determined by difference between *P*_MS_ and CVP. An increase in CVP can result in decrease of CO when it is not associated with concomitant *P*_MS_ augmentation

Acute kidney injury	Increased CVP is associated with increased renal (subcapsular) (interstitial) pressure resulting in decreased renal blood flow, GFR, and derangement in lymph drainage. CVP is a sole hemodynamic parameter that can independently predict the risk of AKI starting from the values above 4 mmHg! In CVP above 15 mmHg, the risk of sepsis-induced AKI exceeds 80%

Splanchnic congestion/and microcirculatory changes [32]	The microcirculation should be recognized as a low pressure part of circulation due to abrupt decrease in blood pressure on the level of resistive arterioles. Therefore, the critical changes in microcirculation have been demonstrated in CVP > 12 mmHg. Any increase in downstream pressure (CVP) results in microcirculation distress

*P*_MS_: mean (systemic) filling pressure, CVP: central venous pressure, and CO: cardiac output.

**Table 3:** Four phases of hemodynamic treatment.

	Rescue	Optimization	Stabilization	Deescalation
Treatment goal	Shock reversal/Life salvage	Adequate tissue perfusion	Zero-to-negative daily fluid balance	Fluid accumulation reversal/edema resolution

Time course	Minutes	Hours	Days	Up to weeks

Hemodynamic targets	Autoregulatory thresholds of perfusion pressure	Micro/macrocirculatory blood flow parameters	Weaning of vasopressors with stable hemodynamic conditions	Return to premorbid/chronic values of pressure and flow

Treatment options	Rapid fluid boluses + vasopressors	Repeated fluid challenges + vasopressors + Inotropes	Maintenance fluids + decreasing/chronic vasoactive agents	Diuretics or other means of fluid removal

## Abbreviations

ACS: Abdominal compartment syndrome
AKI: Acute kidney injury
ANP: Atrial natriuretic peptide
ARDS: Acute respiratory distress syndrome
CO: Cardiac output
CO_2_-gap: Gap between venous and arterial CO_2_ tension
CVP: Central venous pressure
EEO: End-expiratory occlusion test
EGDT/SSC: Early goal-directed therapy/sepsis surviving campaign
EVLW: Extravascular lung water
FRC: Functional residual capacity of the lungs
GFR: Glomerular filtration rate
GIPS: Global increased permeability syndrome
HES: Hydroxyethyl starch
IAP: Intra-abdominal pressure
ICP: Intracranial pressure
ICU: Intensive care unit
ITBV: Intrathoracic blood volume
MAP: Mean arterial pressure
pGDT: Perioperative goal-directed therapy
PLR: Passive leg raising test
*P*_MS_: Mean (systemic) filling pressure
PPV: Pulse pressure variation
ROSD/E: Rescue-Optimization-Stabilization-Deescalation/Evacuation
SAP: Systolic arterial pressure
ScvO_2_: Oxygen saturation on vena cava superior.
